# Helping Mothers and Daughters Talk about Environmental Breast Cancer Risk and Risk-Reducing Lifestyle Behaviors

**DOI:** 10.3390/ijerph17134757

**Published:** 2020-07-02

**Authors:** Carla L. Fisher, Kevin B. Wright, Camella J. Rising, Xiaomei Cai, Michaela D. Mullis, Amelia Burke-Garcia, Dasha Afanaseva

**Affiliations:** 1 UF Health Cancer Center, College of Journalism and Communications, University of Florida, P.O. Box 118400, Gainesville, FL 32611-8400, USA; mullis.md@ufl.edu; 2 Department of Communication, George Mason University, 4400 University, Dr, MSN3D6, Fairfax, VA 22030, USA; kwrigh16@gmu.edu (K.B.W.); crising@gmu.edu (C.J.R.); xcai@gmu.edu (X.C.); 3 NORC at the University of Chicago, 4350 East West Highway, Bethesda, MD 20814, USA; burkegarcia-amelia@norc.org; 4 Fors Marsh Group, 901 N. Glebe Road, Arlington, VA 22201, USA; dafanaseva@forsmarshgroup.com

**Keywords:** breast cancer, environmental risk, interpersonal communication, mother–daughter communication, social media, intervention, lifespan

## Abstract

*Background*: Mothers and daughters struggle to talk about breast cancer risk. Even less attention is paid to environmental determinants of cancer. Third-party online approaches can be helpful navigating these conversations. The aim of this study was to obtain feedback from mothers exposed to a social media intervention (“mommy bloggers”) and identify their preferences for message-design approaches that could help them talk to their daughter(s) about environmental breast cancer risk. *Methods*: We conducted semi-structured interviews with 50 mothers. A thematic analysis was conducted using the constant comparative method. *Results*: Mothers identified four approaches to message design that could help facilitate mother–daughter communication about environmental breast cancer risk. These included two action-oriented approaches that centered on *getting the conversation started* and *keeping the conversation going* and two approaches based on lifespan factors to promote daughters’ engagement by *using age-appropriate language and visuals* and *focusing on developmentally specific lifestyle behaviors*. Mothers also provided recommended strategies within each approach. *Conclusions*: Mothers identified various approaches interventionists can utilize to overcome barriers to talking to daughters about environmental breast cancer risk. To promote mother–daughter communication, the messages should be action-oriented to facilitate interaction, but also developed with lifespan and developmental considerations in mind to engage daughters.

## 1. Introduction

Breast cancer is the leading cause of death for women in the world and the most commonly diagnosed cancer for women living in the United States [1]. Although interventions to reduce breast cancer incidences and promote public health mostly focus on screening, a woman’s risk of developing breast cancer during her lifetime is also linked to lifestyle-related environmental determinants [2]. Unfortunately, environmental linkages with cancer risk can be misunderstood by the public and are not a widely discussed topic among women and their families [3]. Helping families navigate these risk conversations is a critical consideration when developing interventions that aim for women to adopt cancer risk-reducing behavior.

Mothers and daughters with a family history of breast cancer are especially in need of targeted messages to promote risk communication. They may have a heightened risk due to their family’s medical history, and mothers also report significant concerns about their daughters’ risk, especially when the mothers have a personal breast cancer history [4,5,6]. Intervening early in the daughters’ life during windows of susceptibility like adolescence and puberty is crucial to reduce environmental breast cancer risk [7]. The mother–daughter relationship can be instrumental in reducing daughters’ disease risk [4,5,6]. However, mothers and daughters report significant challenges talking about breast cancer risk and healthy lifestyle behavior [4,5,6,8]. They need help learning to facilitate these conversations in a health-promoting manner. Third-party, online, community-based approaches may be ideal in both disseminating environmental risk information and helping mothers and daughters navigate breast cancer risk conversations to promote their adoption of risk-reducing behaviors [6,9,10,11].

As scientists within the National Cancer Institute (NCI) and National Institute of Environmental Health Sciences (NIEHS) co-funded Breast Cancer and Environmental Research Program (BCERP) (see the Supplementary Materials), we formed a partnership with an online community stakeholder group—commonly referred to as “mommy bloggers”—to create and test the efficacy of a social media intervention using a multi-phase design. In Phase 1, we aimed to influence mothers’ risk knowledge and behavioral intentions to reduce environmental breast cancer risk [11]. In Phase 2, we sought to gain critical insight from these bloggers and their readers (i.e., mothers) about the social context (i.e., interpersonal communication dynamics) that influences cancer risk behavior—a key component to developing targeted intervention messages that spark mother–daughter conversations about risk-reducing behaviors.

### 1.1. Reducing Mothers’ and Daughters’ Breast Cancer Risk: Understanding the Social Context

Although interventions tend to focus more on cognitive factors as predictors of behavioral adoption [12], research on cancer and healthy lifestyle behavior that is informed by interdependence theory shows health behavior is embedded within a social context in that our behavior is influenced by relational partners [13]. Interpersonal approaches that target family bonds can effectively promote healthy behavior, including healthy lifestyle habits to reduce cancer risk [9,12,13,14]. Scholarship informed by family systems theory has shown that the way in which families approach health conversations is critical. Productive family communication (e.g., emotional support like listening, openness, shared decision making) can be a key component to facilitating healthy behavior adoption [14,15,16]. For breast cancer risk, mother–daughter communication plays an essential role. Mothers and daughters not only influence each other’s risk-reducing behavior [16,17,18], but the impact of their conversations can be long-lasting for daughters. Research shows that when mothers give daughters risk-reducing breast cancer advice, they tend to follow it into adulthood [17].

Still, both mothers and daughters report that talking about breast cancer risk and engaging in healthy lifestyle behaviors can be distressing [4,5,6,8,18,19,20,21,22]. Younger daughters tend to avoid talking about their risk with their mothers [5,6,19,21] and can even experience a physiological stress reaction during these conversations [8]. Mothers report frustration with their daughters’ avoidance and a heightened concern with reducing daughters’ risk when they themselves have a personal history of the disease [6]. How mothers and daughters navigate these conversations likely plays a role as daughters exhibit higher stress hormones (cortisol levels) during risk conversations if mothers use denial, hostility and antisocial behavior [8]. Families also experience challenges talking about environmental risks of breast cancer. Mothers report difficulty sharing environmental risk information with older and younger generations in the family [23], and some family members may dismiss the information as not relevant to them if they do not have a family history of cancer [24]. They may also find scientific terms associated with environmental determinants (e.g., phthalates) intimidating [24].

Even though disseminating environmental risk information to increase mothers’ awareness is a first step, it is essential that mothers share the knowledge with family members. Mothers have reported that using third-party approaches like magazine articles helps them start conversations with their daughters and also enhances daughters’ comfort level with the topic of risk [2,6]. Studies also indicate that third-party, web-based approaches about environmental factors and breast cancer risk (e.g., videos about radiation risk and BPA/perfluorooctanoic acid) can positively affect mothers’ and daughters’ risk-reducing behavior [9,10]. Third-party approaches accessed online may be ideal as mothers report that the Internet is a primary source of health information, including when seeking breast cancer risk knowledge [25,26,27]. Social media, in particular, has gained in popularity, which allows for a wider reach of mothers and daughters and a more interactive, social experience [28,29].

Bloggers cultivate a rich social environment and can provide a valuable social media platform for disseminating environmental breast cancer risk information and promoting family dialog about healthy lifestyle behaviors [11]. Today in the USA, there are nearly four million female bloggers who are also mothers. This online community often self-identifies as “mommy bloggers” who write “mommy blogs” focusing on topics related to motherhood [29,30,31,32]. Their blogs have been described as a form of community activism [33,34]. Mothers (their readers) report accessing such blogs for both parenting and health-related advice, viewing mommy bloggers as a trusted source in their network [35,36].

Mommy bloggers comprise a community that is not only accepted and trusted by other mothers, but their social media platform provides an opportunity for interventions to have a wider dissemination reach, promoting more mother–daughter communication about risk and healthy behavior adoption. In line with federal encouragement to unite scientists and community advocates to promote healthy behavior, we developed a partnership with mommy bloggers to disseminate a social media intervention on environmental breast cancer risk.

### 1.2. BCERP and Mommy Bloggers Teaming to Promote Healthy Mother-Daughter Behavior

The Interagency Breast Cancer and Environmental Research Coordinating Committee (IBCERCC) was established in 2008 after Congress passed the Breast Cancer and Environmental Research Act. This committee was charged with distinguishing best practices for getting the research into the hands of the public. One best practice identified was to develop community stakeholder-scientist partnerships [37]. In this vein, nearly twenty years ago, NIEHS funded BCERP, which became an exemplary program of scientists and community partners charged with identifying environmental determinants of breast cancer. Over the years, BCERP has translated their research to practice and developed online toolkits that contain brochures, flyers and PSAs, which are available for free on the program’s website [38]. BCERP also capitalized on the influential mother–daughter relationship aiming to intervene early in the lives of daughters by providing a toolkit that was developed specifically for mothers with daughters [38].

**Figure 1:**
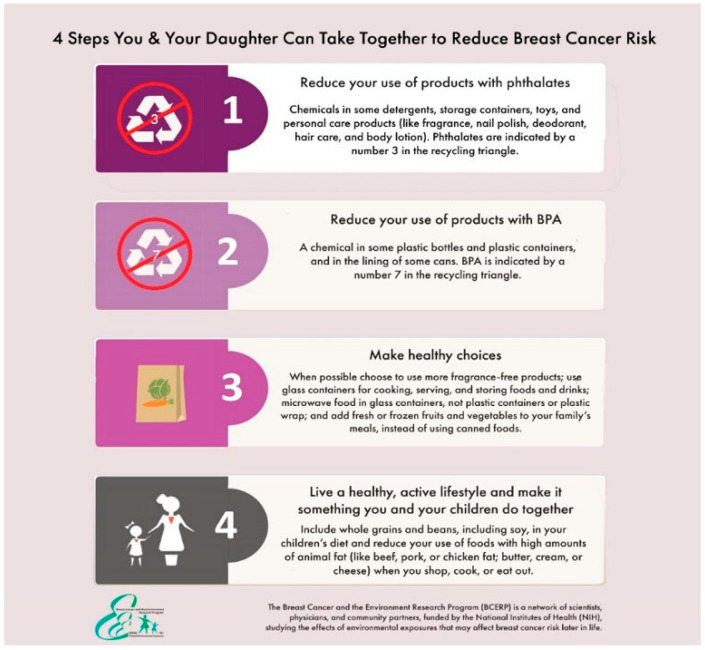
Infographic developed for social media dissemination with mommy bloggers.

To disseminate these materials on social media and further enhance the intervention messages, we used evidence-informed scientific information from BCERP’s toolkit to create a shareable infographic (see Figure 1). The image offered mothers and daughters a four-step approach to making lifestyle changes together in an effort to reduce their environmental risk of breast cancer. We teamed with a diverse group of mommy bloggers and asked them to write a blog about environmental breast cancer risk in a way that would appeal to their readers that also included the infographic, an un-editable paragraph about BCERP to identify the scientific source of the information and a link to BCERP’s full toolkit (available here: https://bcerp.org/educational-materials/materials-for-parents-and-caregivers/). We successfully disseminated the information, reaching more than 400 mothers, and showed the efficacy of this communication channel in promoting public health. The intervention had an impact on mothers’ risk-reducing knowledge, intentions to engage in risk-reducing behavior and likelihood that they shared the information with their daughters [11].

In the second phase of the study, reported herein, we aimed to further refine BCERP’s mother–daughter toolkit in a way that promoted mother–daughter communication about risk and health behavior changes. The aim of this study was to obtain feedback from mothers previously exposed to the intervention (bloggers and readers) to identify mothers’ preferences for message-design approaches that could help them talk to their daughter(s) about environmental breast cancer risk and promote the adoption of risk-reducing behavior (i.e., lifestyle changes).

## 2. Materials and Methods

### 2.1. Sampling and Recruitment

To obtain feedback from bloggers and mothers exposed to the intervention, once the intervention period ended, we began Phase 2 using an interpretive design and constituent-involving approach to recruit mothers for a semi-structured interview. This design allows us to bring mothers’ voices to the forefront in identifying message-design approaches that can promote mother–daughter communication and healthy behavior adoption. This approach is also in line with recent calls in health disparity research to address more than only biomedical or psychological constructs of the social context of health behavior [39].

Once we obtained IRB approval, we used purposive sampling to recruit a racially and ethnically diverse group of bloggers and readers exposed to the social media intervention [11]. We broadened our reach to mothers without daughters to promote diversity, although nearly all mothers who did participate had at least one daughter (84%). A research coordinator helped with recruitment and scheduled interviews with 50 women (40 bloggers, 10 readers; M age = 39). These mothers were racially and ethnically diverse with 40% White or Caucasian, 30% Black, 22% Hispanic and 8% Other (Asian, Middle Eastern, mixed race). The majority had a family breast cancer history (70%) and a college degree (77%).

### 2.2. Procedures and Analyses

Prior to the interview, informed consent was obtained. Interviews were audio-recorded and conducted by phone. Although women had been exposed to the intervention materials during Phase 1, women were emailed all materials again (infographic, documents in the BCERP toolkit, and online link) to ensure they would be readily available for the interview. During the interview, mothers were asked to share experiences talking about breast cancer and environmental risk with their family, particularly their daughters (e.g., *What should we know about your experiences when sharing the information with your family or making lifestyle choices?).* They were also asked for feedback on how to improve the toolkit to enhance their ability to interact with their daughters about the topic and promote healthy behavior engagement together. Interviews were professionally transcribed for words spoken (633 pages of data).

A thematic analysis was conducted according to steps outlined in the constant comparative method [40,41]. Two authors on the team conducted 50% of interviews over a two-week period. Operational and analytical memos were kept and discussed after interviews to identify patterns, refine the script and develop an initial codebook paying specific attention to approaches mothers perceived would help them talk to their daughters about breast cancer and environmental risk-reducing behavior. Two additional authors helped with the rest of the interviews until data saturation was attained. Using the initial codebook, one author constantly compared and analyzed all transcripts by racial and ethnic groups to ensure any notable differences could be captured and confirm thematic saturation. Early on patterns emerged across all groups of mothers allowing us to identify approaches and strategies that can resonate with more mothers. An additional 10 interviews were conducted and analyzed to confirm thematic saturation and ensure validity of the analysis. Once analysis was completed, the lead qualitative expert on the research team reviewed the analysis and conducted axial coding. This allowed us to further define each theme (the message design communication approach) by identifying thematic properties (the message design communication strategies) that mothers identified within each approach.

## 3. Results

Mothers across races and ethnicities identified four approaches to message design that could help facilitate mother–daughter communication about risk and their engagement in risk-reducing lifestyle changes. Mothers described two action-oriented approaches that centered on *getting the conversation started* and *keeping the conversation going*. They also identified two approaches to personalize the messages (based on lifespan factors) to promote communication and daughters’ engagement by *using age-appropriate language and visuals* and *focusing on developmentally specific lifestyle behaviors*. Each theme (or message design communication approach) was characterized by thematic properties (identified in italics below). Those thematic properties represent message design communication strategies that mothers identified within each message design approach they perceived would enhance their ability to talk to their daughters about environmental breast cancer risk. Mothers’ authentic narratives elevate their voice in helping interventionists better target environmental risk messages in ways that promote mothers’ and daughters’ adoption of risk-reducing lifestyle behaviors. Findings are presented in Table 1 as action statements using the ecological sentence synthesis approach, which helps to facilitate the translation of findings into practice by interventionists [42,43]. Pseudonyms are used in the presentation of findings to maintain participants’ confidentiality.

### 3.1. Getting the Conversation Started

Mothers stressed the need for the information to include action-oriented components to better understand how they could spark these mother–daughter discussions about breast cancer and environmental risk. They identified three strategies for getting the conversation started: (1) *include conversation prompts,* (2) *integrate family-centered activitie*s and (3) *include an online interactive tool*.

Mothers described the need to *include conversation prompts* to help them start conversations with daughters. Mothers’ suggested “prompts” include “skits and examples of what you should say,” “a list of questions,” “talking points,” and “a back and forth script” for mothers and daughters to work through. Mothers viewed these prompts as a “how to” tool to initiate the conversation. As one mother explained, “I think having dialogue to where you sit down with your children and you have the answers in front of you where they can ask these questions and you’re not scrambling” (Lissy). Mothers stressed that this action component was necessary as the topic itself (cancer risk) could be challenging to navigate.

Additionally, mothers wanted the information to *integrate family-centered activitie*s to help them start the conversation and better ensure they could initiate lifestyle behavior changes together. Mothers perceived that activities made the information more learning-focused, interactive and fun (or more like a “game”), which would enhance daughters’ willingness to engage in the topic. Mothers suggested various activities like making the behavior change a “challenge” (i.e., an online social movement) or including a mother–daughter activity to do an at-home “scavenger hunt” like “an inventory almost of ‘Check your house for these things!’” (Samira). Mothers also recommended including a “printable grocery list” or an activity that tasked mothers and daughters with doing a scavenger hunt in a store to identify products made with phthalates or BPA, which would in turn help them find healthier brands to purchase in the future. As one mother, Tasha, stated, “design [it] for kids to be able to pick out and find the healthy versus unhealthy [thing] or the thing they want in their body versus what they don’t want.” Similarly, mothers advised including healthy recipes for creating safe household products (e.g., cleaners) or healthier meals they could make together. As Annie stated, “We could make strawberry and spinach smoothies and just show them something about how your body loves good stuff and why fresh is best.”

Mothers also suggested a strategy to *include an online interactive tool* that would help them facilitate the conversation about risk with their daughters. Like activities, they described this approach as a way to bring them together in a more interactive, learning-centered manner. As one Monique suggested, an online tool could be “something that rewards them for remembering to do things … and you accumulate points or something that rewards them for doing the right thing.” Mothers described seeing and using an interactive tool on other websites that helped them start talking to their daughters about other challenging topics like puberty. As Cally shared,

If there was something I could sit with my kids and go through with them and they could click … like an interactive toolkit maybe. They could help me click through something that would show what can we do in this situation. … Is this important? Is that important? Something we could maybe [do] together instead of reading to the kids.

### 3.2. Keeping the Conversation Going

In addition to getting the conversation started, mothers wanted an action-focused approach to help them keep the conversation going. They stressed the importance of maintaining discussions with their daughters about breast cancer risk over time. They identified two strategies to message design aimed at sustaining mother–daughter communication: (1) *incorporate a multipronged, longitudinal approach* and (2) *provide reminders.*

Mothers suggested dissemination *incorporate a multipronged, longitudinal approach.* They recognized that the bulk of breast cancer risk information is disseminated once per year, typically during October—a time in which information about risk is “competing with” so much other breast cancer information. As Makayla observed, “people will tune out if they see the same message over and over.” Mothers suggested instead to disseminate information across the year during other relevant times and to simultaneously use multiple social media outlets to keep the conversation going (e.g., Facebook, bloggers, news). For instance, mothers talked about making lifestyle changes with their daughters during the New Year with their resolutions. They also noted that the information was relevant to “spring cleaning” in the household with the turn of seasons, a time in which they could together clear out unhealthy products and adopt healthier options. They also suggested providing a monthly action plan for mothers and daughters, thereby providing a feasible way for them to enact the four-step approach portrayed in the infographic (see Figure 1) over time.

Relatedly, mothers explained the importance of dissemination that could *provide reminders* or “jolts” to keep the information fresh (addressing memory) and more at the forefront of their lives (accommodating busy schedules). As Kinsey stated, “We’re so busy, busy, busy with doing our everyday things that we forget.” Reminders included tangible tactics that mothers and daughters could together reference and “be reminded of (making) better choices” (Melanie). For instance, mothers suggested including daily reminders like a magnet of the four-step image that they could see daily on their refrigerator or bathroom mirror.

### 3.3. Using Age-Appropriate Language and Visuals

Mothers indicated that the information needed to be more relatable or personalized for daughters to promote mother–daughter communication and encourage daughters’ interest. Mothers noted that lifespan factors were important and identified three strategies for personalizing messages in this manner: (1) *use words daughters understand,* (2) *reduce or replace “scare factor” terms with lifestyle terms,* and (3) *incorporate fun, age-appropriate visuals.*

Mothers wanted the information to *use words daughters understand.* They noted that the language needed to be different “depending on (daughters’) age and even maturity level” to ensure comprehension. As Kari explained, “Put it into words that my kids could understand.” Mothers with adolescent daughters acknowledged that this was particularly important given they may receive incorrect information from peers. Mothers with daughters in childhood suggested not using scientific or technical terms. As Natalia explained,

I wouldn’t tell my 6-year-old to reduce her use of products with—I can’t even say the word! “Phthalates?” … When you’re talking to younger kids, you need to talk at their level. … That stuff about chemicals in detergents and toys. … Maybe put a toy up there. You have to speak their language and break it down simply, “Some of these things aren’t good for you. There might be stuff in my laundry detergent and some in your toys.”

Mothers also suggested the information should *reduce or replace “scare factor” terms with lifestyle terms.* They advocated for an approach that was less disease specific and more focused on lifestyle habits or “something that we need to do to take care of our bodies” (Tasha). For instance, mothers preferred language like “healthy habits,” “healthy living,” or “healthy choices” instead of disease-specific terms like “cancer” or “risk.” Mothers with younger children wanted to use the term “germs” to replace “chemicals” or “cancer.” Although mothers noted comprehension was always a concern, they mainly focused on the “scare factor” of certain terms, particularly disease-specific words like “cancer.” If daughters were scared (or if mothers did not want to scare them), this could shut down communication about breast cancer risk behavior. Some mothers suggested eliminating the word “cancer” altogether and instead focus on healthy choices. Olivia explained why:

I’m more about examples with my daughter: “You see that mommy doesn’t do these things (microwave in plastic) so you don’t do these things” Versus saying that you might get cancer if you drink out of BPA bottles. We are more focusing on just living a healthy lifestyle in general versus let me scare you with these words. … I don’t want them to develop anxieties around different things.

Mothers noted that these language choices had to be considered in terms of the daughter’s age and maturity as well as behavioral cues. As Cally, a mother of an early adolescent daughter stated,

She does ask a lot of questions and I know that she understands and seems interested. … Just approaching them honestly and having a conversation about it and don’t scare them and say “Oh no—this stuff happens” because there’s so many things that could happen.

Mothers also expressed that the information disseminated should *incorporate fun, age-appropriate visuals* to engage daughters. They believed that this strategy could lighten the tone and make the information more enjoyable. Mothers with daughters in childhood or early adolescence wanted more kid-friendly displays of information so that they could share it with their daughters, like graphics, cartoons, brighter colors or characters children recognized. Mariana had similar suggestions for “tweens:”

Graphics that look more kid friendly—just stick figures with pigtails or something like that. … Graphics that kids could kind of relate to would be helpful like “I’m going to encourage mom to buy vegetables that are frozen or fresh and not in a can.”

At times, this strategy also included a more action-focused approach to personalizing the information in line with the daughter’s age. For example, one mother referenced the color-coded charts for behavior monitoring that are commonly used in elementary schools as a similar approach to use with children making healthier choices at home to acknowledge and reward behavior change (e.g., okay–better–best system of lifestyle choices). Likewise, mothers with teenage daughters described the importance of visually communicating information in other social media platforms. They recognized that daughters in this age range used many “how-to” videos on Instagram and YouTube. They perceived that this visual strategy could draw daughters in to learn healthy habits while also potentially promoting healthier products or brands.

### 3.4. Focusing on Developmentally Specific Lifestyle Behaviors

Mothers noted that another way to personalize the risk messages was by focusing on developmentally specific lifestyle behavior relevant to daughters at different phases of the lifespan. This approach was interrelated to strategies for starting the conversation as well as keeping the conversation going. Mothers offered two strategies for making the information developmentally relevant: (1) *integrate all phases of human development* and (2) *include products specific to daughters’ developmental needs.*

Mothers suggested that the messages *integrate all phases of human development* on some level. They believed this provided information that would allow for an ongoing discussion of how risk and healthy behavior were relevant across the lifespan (e.g., from pregnancy to birth to puberty, etc.). It also ensured that mothers could access information and, thus, start the conversation with risk-reducing information that was actually relevant (or of interest) to the daughters based on their age. Antonia explained this strategy while referencing images in the toolkit:

You have everything from pregnancy to baby to elementary age. … (Maybe) breaking up into where you have a self-focus of, okay, “You’re pregnant. This is what you should consider.” Or okay, “Your child is in their toddler years, elementary years.” Preteen might be a little bit more pinpointed.

In addition, mothers suggested the information *include products specific to daughters’ developmental needs.* Mothers noted that daughters were interested in variant products at different points in the lifespan. As Sydney stated, “(going) in that direction where the things that they love (that) hits home immediately.” Mothers believed this strategy would appeal to daughters’ interests and promote their willingness to talk while helping them learn how to make healthier product choices (i.e., phthalate- or BPA-free). For instance, daughters in childhood were interested in toys as well as nail polish or lip balms and gloss. Mothers stated that preteens and teenaged daughters were into nail polish, body lotions, shampoos and body washes, hair products, fragrances, deodorant, as well as lip gloss and makeup. As Antonia shared,

She’s getting to that age where she wants to spray herself with everything all the time. That might be a good starting point. It would be easy to go there with her and we can talk about what it said and then how we can do it in our own life. … There are some products that are going to be more appealing based on age.

Mothers indicated that this was especially important for adolescent daughters who were starting to learn more about their bodies and make independent lifestyle choices. As Elena explained,

Teenage girls are starting to wear deodorant and body lotions. And if they’re working or collecting allowance, they’re at the age where they have some kind of independence and they’re buying their own products. But if you start teaching young girls to look for the ingredients and say, “When you’re buying a lotion or deodorant or lip gloss, look out for these ingredients. And if you see something with these ingredients, maybe not purchase it.” … (Learning) how to read the label of personal care items so that they can make better decisions (versus) “If it smells nice I was going to get it!”

## 4. Discussion

The results provide message design approaches and strategies—identified by mothers—that interventionists can incorporate when designing environmental breast cancer risk messages to help facilitate mother–daughter interaction about changing their health behavior. Previous scholarship has revealed that mothers find talking about breast cancer risk challenging. Daughters avoid such conversations, and mothers’ communication approach to the topic can contribute to daughters’ distress [4,5,6], potentially resulting in a physiological stress response from daughters [8]. Third-party, online approaches (such as the social media intervention) [11] can help mothers introduce these topics. Mothers in this sample identified various approaches interventionists can utilize that may aid mothers and daughters in overcoming these barriers to talking about environmental breast cancer risk.

Mothers’ feedback illustrated the importance of ensuring message-design approaches are not only knowledge- or learning-focused, but also action-oriented. To encourage mother–daughter communication, mothers need messages that help them both prompt and sustain interaction about cancer risk and lifestyle behaviors. As a mother in our sample observed, the “toolbox” needs to actually include “tools” for talking about environmental breast cancer risk. These mothers offered a wealth of interaction-promoting strategies or “tools” to include in message design, such as providing prompts or scripts to help mothers and daughters get the conversation started, mother–daughter activities like scavenger hunts and recipes to facilitate shared learning about environmental determinants and healthy lifestyle choices, and online tools or reward systems to encourage dialogue as well as behavior adoption.

Mothers’ suggestions also highlight the need for a more personalized approach to risk information dissemination with more targeted messaging based on daughters’ unique needs and interests. To promote mother–daughter communication, the messages should be developed with lifespan and developmental considerations in mind. Mothers’ feedback about lifespan factors is especially critical to ensuring daughters are not only interested, but comfortable talking about risk (and less likely to avoid these conversations). To date, dominant theories used in developing health and risk messages do not incorporate a lifespan perspective [44]. Mothers’ insight helped to show how a lifespan approach may enhance the persuasive appeal of environmental breast cancer risk messaging and, ultimately, be an imperative factor in facilitating critical mother–daughter communication about engaging in risk-reducing behavior.

### A Lifespan Approach to Environmental Breast Cancer Risk Interventions

Lifespan scholars have demonstrated that communication is fundamentally developmental, and that human behavior can only be understood with developmental factors in mind [44,45,46,47,48]. This includes age and maturity differences, sociohistorical differences in communication norms and preferences, life stage variation in goals and concerns, as well as intergenerational communication dynamics such as those that characterize mother–daughter bonds [6,44,45]. Yet, dominant theories that inform health message design aimed at persuading people to engage in healthier behavior (e.g., the health belief model, theory of planned behavior) are not informed by a lifespan perspective or focused on developmental factors.

Leading lifespan theorists have recently advocated for more consideration of developmental variables in health intervention initiatives:

A logical extension of the lifespan communication perspective is to question whether health and risk messages and risk communication directed toward individuals of differing ages need to account for the fundamental developmental differences that exist within and around individuals of different ages.

*[44]*

Renowned researchers in cancer risk message design have acknowledged that “targeted” messages often are not as “individualized” as they could be, noting the need for audience segmentation (more targeting) as well as, ideally, individualized or personalized approaches [49]. However, they also affirm that individualized approaches (i.e., tailored messaging specific to one individual) are not typically feasible and realistic in health interventions [49]. Our results support the notion that a lifespan approach can enhance interventionists’ ability to segment the audience further and design more targeted or personalized messages (messages that are more likely to appeal to daughters) based on developmental considerations.

Mothers in this study stressed that messages target the daughter’s developmental phase in terms of both content and dissemination. This was critical for mothers to start the conversation and maintain it across the lifespan of their daughter’s development. This included targeting the messages in terms of incorporating age-appropriate language (e.g., ensuring they understand the information), age-driven visuals (e.g., including cartoons to appeal to daughters in childhood) and developmentally focused activities of lifestyle choices (e.g., having scavenger hunts to identify safe products tied to products that piqued daughters’ interests according to their age). To engage their daughters, the information needs to be both relevant and useful based on daughters’ developmental maturity and associated interests. Moreover, mothers indicated that the information should be developmentally appropriate in that it was “not scary,” particularly with younger daughters. Previous research indicates that daughters avoid talking to their mothers about risk and exhibit discomfort when doing so [4,5,6,8]. Mothers in our study expressed concerns about their daughters being scared or anxious with disease-specific terms, offering substitutions that may enhance their comfort (e.g., focusing on lifestyle habits or germs instead of “cancer”). Considering their maturity or age may be especially critical to ensuring daughter’s comfort level.

Mothers also stressed the need for interventionists to consider the lifespan phase of their audience when developing and timing dissemination of health behavior messages. For example, mothers wanted a more longitudinal approach to receiving breast cancer risk information, expressing that the primary time in which they receive this knowledge is in October (breast cancer awareness month). Mothers believed that this time period is not ideal given the overload of information during that short time period in addition to the busyness of their lives as midlife mothers. Mothers advocated for a more longitudinal, multipronged dissemination approach (with reminders), but also specified other time periods in which the information—particularly about environmental health determinants and lifestyle behavior—is relevant (e.g., during New Year’s or traditional spring cleaning time periods). Additionally, mothers stressed that the dissemination channel be chosen in consideration of daughter’s generational cohort preferences for receiving information. They noted that while children may appreciate a more animated, study-based cartoon that portrayed the information, adolescent and teenage daughters gravitated toward social media like YouTube videos, which have become a forum for teens to learn behavior (e.g., how to apply makeup). For this age group of daughters, mothers (many of whom as mommy bloggers were themselves social media professionals) noted the value of disseminating this information via a social media platform that the daughters would actually be interested in.

In summary, mothers’ suggested approaches and strategies outline the critical role lifespan or developmental factors play not only in environmental breast cancer risk information, but in moving mothers and daughters to engage in risk-reducing behaviors. Interventionists may benefit from what lifespan theorists have recently advocated for in terms of advancement in health and risk message design. By incorporating a lifespan communication lens, interventionists may be able to create more “age sensitive” messages and, thereby, increase their persuasive effectiveness [44]. At the same time, by having a lifespan approach to health messages design and intervention dissemination, the conversation about cancer risk and risk-reducing behavior can be integrated across the entirety of women’s and girls’ lives.

## 5. Conclusions

Mothers provided valuable insight on message design approaches and strategies to disseminating information in a way that could both initiate and sustain mother–daughter communication about environmental breast cancer risk and adopting healthy lifestyle behaviors. Developing habits is a time-sensitive experience. Future research is needed to test the various approaches and strategies aligned with various age groups of daughters as well as evaluate the impact of the intervention over time. Testing approaches that ease daughter’s comfort and decrease their avoidance of the topic would be prudent.

Collectively, our findings provide a set of message design approaches to further target messages to mothers and daughters. Given our diverse sample, these approaches and strategies may speak to mothers and daughters across racial and ethnic groups. However, communication preferences and expectations among mothers and daughters do vary by culture [50]. Moreover, across cultures, privacy can be a barrier to talking about health for older generations who are more socialized to not share health information [23]. Privacy concerns may be even more heightened when messages are disseminated digitally. Future message testing should evaluate the impact of these approaches and strategies on various health outcomes (e.g., knowledge, behavioral intention, mother–daughter interaction, adoption of healthy behaviors, reduction of cancer incidence) while being mindful about how particular message design approaches and strategies, including social media dissemination, may be more influential or preferred among particular groups of mothers and daughters.

## Figures and Tables

**Table 1:** Message-design approaches and strategies to promote mother–daughter communication about environmental breast cancer risk.

**Messages Should Be Action-Oriented and Help Mothers in**	**Using These Strategies**
getting the conversation started	conversation prompts (e.g., a list of Q&A’s, skits, talking points) family-centered activities (e.g., scavenger hunts, recipes, challenge) an online interactive tool (e.g., to track behavior rewards, to click through together)
keeping the conversation going	a multipronged, longitudinal approach (e.g., disseminate messages across the year during salient behavior change times like New Year’s or spring cleaning, include a monthly action plan) reminders (e.g., disseminate reminders for information recall, include daily reminders like magnets)
**Messages Should Be Personalized Using Lifespan Factors and Appeal to Daughters by**	**Using These Strategies**
using age-appropriate language and visuals	words daughters can understand (e.g., age-level wording, simply stated information) avoiding “scare factor,” disease-specific terms (e.g., instead of “cancer” use lifestyle terms like “healthy habits”) fun, age-appropriate visuals (e.g., brightly colored cartoons for kids and how-to YouTube videos for teens)
focusing on developmentally specific lifestyle behaviors	integrating all phases of human development (e.g., make it specific based on age and relevant across the lifespan) including products specific to daughters’ developmental needs (e.g., focus on skin and hair products for teens)

## Supplementary Materials

BCERP’s web site for the full toolkit: https://bcerp.org/educational-materials/materials-for-parents-and-caregivers/.
